# Whole genome sequencing reveals large deletions and other loss of function mutations in *Mycobacterium tuberculosis* drug resistance genes

**DOI:** 10.1099/mgen.0.000724

**Published:** 2021-12-10

**Authors:** Laura C. Gomes, Susana Campino, Cláudio R. F. Marinho, Taane G. Clark, Jody E. Phelan

**Affiliations:** ^1^​ Department of Parasitology, Institute of Biomedical Sciences, University of São Paulo, São Paulo, Brazil; ^2^​ Faculty of Infectious and Tropical Diseases, London School of Hygiene and Tropical Medicine, Keppel Street, London WC1E 7HT, UK; ^3^​ Faculty of Epidemiology and Population Health, London School of Hygiene and Tropical Medicine, Keppel Street, London WC1E 7HT, UK

**Keywords:** antimicrobial resistance, WGS, tuberculosis

## Abstract

Drug resistance in *Mycobacterium tuberculosis *, the causative agent of tuberculosis disease, arises from genetic mutations in genes coding for drug-targets or drug-converting enzymes. SNPs linked to drug resistance have been extensively studied and form the basis of molecular diagnostics and sequencing-based resistance profiling. However, alternative forms of functional variation such as large deletions and other loss of function (LOF) mutations have received much less attention, but if incorporated into diagnostics they are likely to improve their predictive performance. Our work aimed to characterize the contribution of LOF mutations found in 42 established drug resistance genes linked to 19 anti-tuberculous drugs across 32689 sequenced clinical isolates. The analysed LOF mutations included large deletions (*n*=586), frameshifts (*n*=4764) and premature stop codons (*n*=826). We found LOF mutations in genes strongly linked to pyrazinamide (*pncA*), isoniazid (*katG*), capreomycin (*tlyA*), streptomycin (e.g. *gid*) and ethionamide (*ethA, mshA*) (*P*<10^−5^), but also in some loci linked to drugs where relatively less phenotypic data is available [e.g. cycloserine, delaminid, bedaquiline, *para*-aminosalicylic acid (PAS), and clofazimine]. This study reports that large deletions (median size 1115 bp) account for a significant portion of resistance variants found for PAS (+7.1% of phenotypic resistance percentage explained), pyrazinamide (+3.5%) and streptomycin (+2.6%) drugs, and can be used to improve the prediction of cryptic resistance. Overall, our work highlights the importance of including LOF mutations (e.g. large deletions) in predicting genotypic drug resistance, thereby informing tuberculosis infection control and clinical decision-making.

## Data Summary

All sequencing data used in this study wre downloaded from the EBI SRA (https://www.ebi.ac.uk/ena/browser/home). All accessions are listed in Data S1 (available in the online version of this article).

**Impact Statement**

Antimicrobial resistance has increasingly become a roadblock towards the goal of tuberculosis elimination. Whole genome sequencing (WGS) of *Mycobacterium tuberculosis* can be used to predict resistance by scanning for known SNPs, but large deletions are often overlooked in bioinformatic pipelines. The importance of large deletions is currently not well understood with just a few sporadic reports of deletions causing resistance in the literature. This work presents the first large-scale characterization of the prevalence of resistance causing large deletions and other loss of function mutations in a large dataset of over 32000 isolates with WGS data. We find that large deletions account for a large proportion of resistance-causing mutations for drugs such as *para*-aminosalicylic acid, pyrazinamide and streptomycin. This work will inform the design of bioinformatic pipelines developed by research and public health institutions, to perform sequencing-based predictions of drug resistance for epidemiological and infection control applications.

## Introduction

Tuberculosis disease (TB), caused by members of the *Mycobacterium tuberculosis* complex (MTBC), poses a major burden on health globally, with 10 million new cases and 1.4 million deaths worldwide in 2019 [1]. TB is transmitted through aerosol particles that are inhaled into the lungs. TB is treated with antibiotic drugs, usually a standard course consisting of 6 months of isoniazid and rifampicin, supplemented with ethambutol and pyrazinamide in the first 2 months. Treatment success rate for TB has been estimated at 85% globally [1], but is reducing due to increasing drug resistance, with an estimated 56% success for multi-drug-resistant TB (MDR-TB) [1]. MDR-TB is defined as resistance to both isoniazid and rifampicin, and requires supplementation with second-line antibiotics such as fluoroquinolones and aminoglycosides. Resistance to second-line drugs can also develop leading to extensively drug-resistant TB (XDR-TB). Half a million people developed MDR-TB in 2019, with XDR-TB present in at least 60 countries, and it is estimated that only one in three people who developed drug-resistant TB had access to an appropriate drug regimen [1].

Resistance to antibiotics in *M. tuberculosis* arises from mutations in its genome. SNPs and insertions or deletions (indels) can arise in genes coding for drug targets or pro-drug activators [2, 3]. These mutations disrupt the interaction of the drug with the translated proteins, leading to resistance. SNPs are the most common form of variation leading to resistance and often occur in regions of proteins involved in drug interactions [4, 5]. Small indels occur often in non-essential genes coding for pro-drug activators (e.g. *pncA*) leading to frameshifts and loss of function of the protein [2, 6]. Indels have also been found in essential genes coding for drug targets such as *rpoB*, where only in-frame indels are observed and, thus, function is preserved [4, 7]. Less well investigated is the role of large deletions as a mechanism of resistance. Large deletions have been previously observed in non-essential pro-drug activators such as *pncA* [8] and *thyA* [9], but their contribution to global drug resistance is currently unknown. Frameshifts, premature stop codons and large deletions all cause major alterations to the size and sequence of the encoded protein and can be collectively termed as loss of function mutations (LOF) owing to the normal biological function of the protein being disrupted.

Whole genome sequencing (WGS) has steadily decreased in cost since the introduction of next generation sequencing (NGS) technology [10]. NGS platforms allow for the characterization of a range of genome-wide variants, including larger structural variants such as large deletions. WGS has been widely used to investigate the emergence of drug resistance in *M. tuberculosis* [11–13]. Variants identified in WGS data have been used to predict ‘gold standard’ phenotypic resistance in cultured *M. tuberculosis* with a high sensitivity and specificity [11, 14, 15]. Genotypic resistance characterization to inform clinical and infection control decision-making has already been adopted in high-income low-burden settings such as the UK and the Netherlands, but will be of most benefit in high-burden TB settings [15–17]. Several tools have been developed to profile WGS data to predict drug resistance [14]. However, due to the added complexities of finding and evaluating deletions together with a lack of evidence on their prevalence, detection of large deletions is often omitted from WGS data pipelines. Indeed, in the initial papers detailing several tools for profiling resistance in *M. tuberculosis *, only two of eight mention the calling of large deletions [18–25]. Here, we characterize the contribution of LOF mutations, including large deletions, frameshifts and premature stop codons, to drug resistance in more than 32000 *M *. *tuberculosis* clinical isolates with associated phenotypic data.

## Methods

Raw sequence data were downloaded from the European Nucleotide Archive (ENA) (see Data S1 for accession numbers). Samples were only considered if they had: (1) no evidence of mixed infections based on TB-Profiler [14] lineage prediction; (2) >90% reads mapping to the genome; (3) >30-fold average coverage; (4) >95% of the genome with at least 10-fold sequencing coverage. Processing of sequence data was performed using the same software and pipeline as used in TB-Profiler to find small variants and large deletions. In short, sequence reads were trimmed using trimmomatic [26] (v0.39) using the parameters LEADING:3 TRAILING:3 SLIDINGWINDOW:4:20 MINLEN:36. Trimmed reads were aligned to the H37Rv reference genome (AL123456.3) using bwa-mem [27] (v0.7.17) software with default parameters. Small variants were called using freebayes [28] (v1.3.2, --haplotype-length 1) software. Variants were filtered to remove those that were supported with <10 reads coverage. Stop gained and frameshift mutations were retained for subsequent analysis if occurring within the first 95% of the gene length. Delly software (v0.8.1) [29] was used to find indels from the WGS data. Analysis was restricted to robust variants, namely large deletions (using the -d DEL option), as well as small indels that overlapped with those found by GATK, Samtools and freebayes software tools. Indels were filtered to retain only those matching the following criteria: (1) >70% read pairs and split reads supporting the alternate allele; (2) between 50 bp and 50 kbp in length; and (3) overlap with the coding regions of drug resistance genes. All indels were checked manually using the IGV genome browser to assess the quality of sequence data alignments. Association analysis was performed using logistic regression implemented in the ‘statsmodels’ python package [30], allowing the estimation of odds ratios and *P*-values. Sample (sub-)lineage was used as a covariate to account for potential population structure effects. *P*-values were adjusted using a Bonferroni correction to account for type I error inflation. Drug susceptibility testing phenotypes used for the association analysis have been previously collated from the literature (see [14] for details). Variant counts were aggregated at a gene level to ensure a robust association analysis of rare mutations [4]. The TB-Profiler [14] tool was used to *in silico* predict drug resistance and identify variants in 42 established resistance loci (github.com/jodyphelan/tbdb).

## Results

**Fig. 1.**
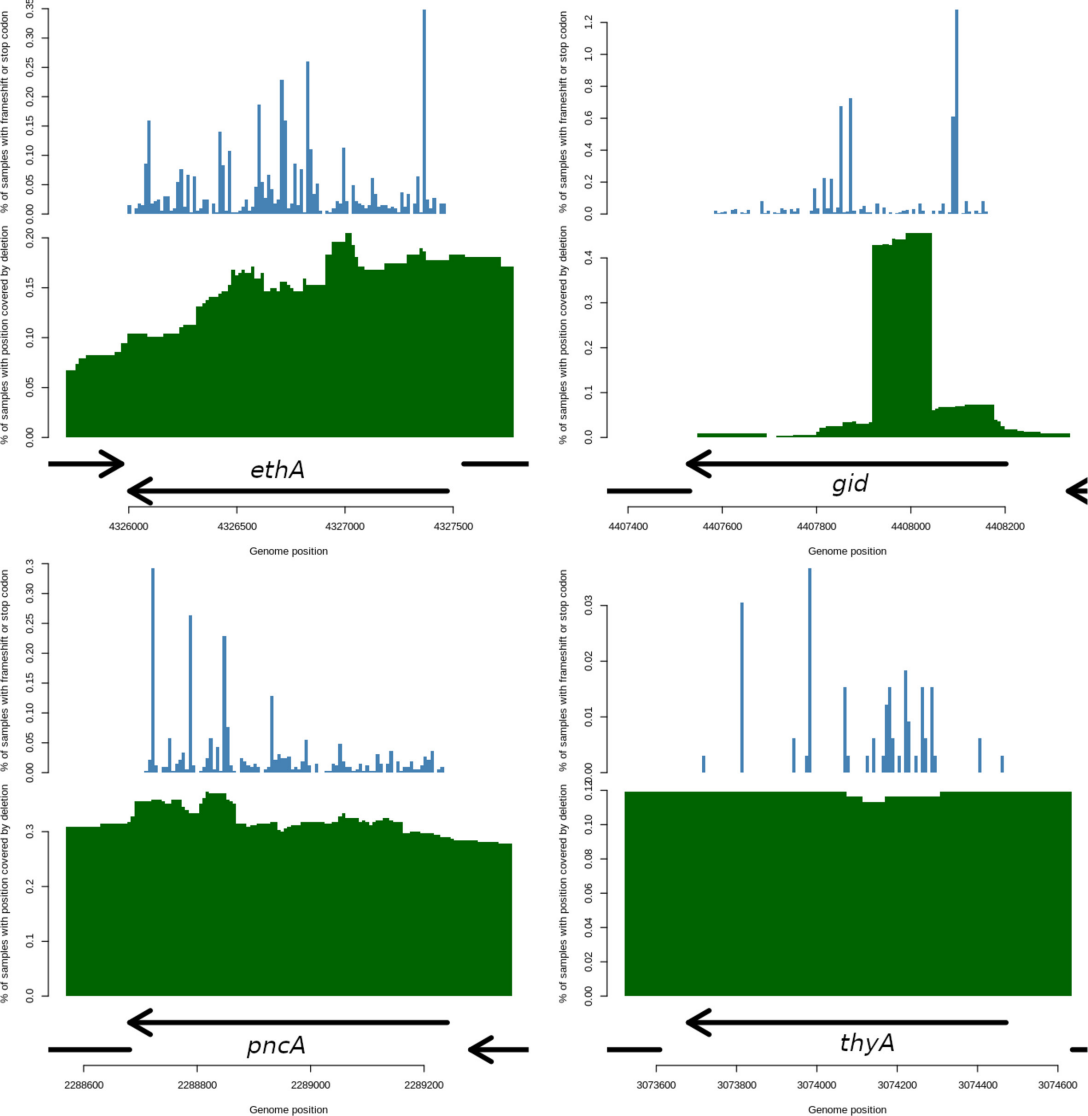
Maps of loss of function mutations for *ethA*, *gid*, *pncA* and *thyA*. Arrows show the genomic location of the gene with the direction indicating whether it is on the positive or negative strand. The upper panel (blue) shows the location of frameshifts and premature stop codons across the gene with the *y*-axis representing the percentage of isolates which have either of these variants. The variants are scattered throughout the gene with no clear clustering observed. The lower panel (green) represents for each position the percentage of samples which have an overlapping large deletion. Interestingly, a large peak is observed in *gid* and represents a large number of isolates with an identical deletion.

Raw sequence data for 32689 *M *. *tuberculosis* samples was aligned to the H37Rv reference genome and led to an average coverage of 93-fold (range: 30–3951). The majority of samples covered lineages 1 to 4 (L1 9.7%; L2 25.3%; L3 11.5%; L4 51.1%), were predominantly pan drug-susceptible (62.8%) (Table S1) and covered all TB endemic geographical regions (Fig. S1). High numbers of drug-resistant strains (37.2%) were present (pre-MDR or MDR 22.0%, pre-XDR or XDR 9.8%, other 5.5%) (Tables S1 and S2). All downstream analysis focused on 42 drug resistance candidate genes used to predict resistance in TB-Profiler [14]. Across the 42 drug resistance genes, counting of variants (aggregating the number of times a variant appears across all samples) led to 280492 high-quality mutations being identified, including 274549 non-synonymous SNPs (8904 unique), 5357 small indels (1031 unique; size: median 1 bp, range 1–96 bp) and 586 large deletions (271 unique; size: median 1115 bp, range 52–39725 bp). Large deletions were found across 14 genes, with frequencies varying from a single occurrence in *embB* (ethambutol) to 165 occurrences in *gid*. The genes with the highest number of deletions were *gid* (165, streptomycin), *pncA* (158, pyrazinamide), *ethA* (72, ethionamide), *katG* (50, isoniazid) and *thyA* (41, PAS) (Table S3, Fig. 1). The remaining nine (from 14) genes had relatively fewer instances (range 1–29).

Other LOF mutations (non-large deletions) were identified in 23 of the 42 drug resistance genes, and included frameshifts (*n*=4764, 834 unique) and premature stop codons (*n*=826, 187 unique). The number of such mutations found differed greatly, ranging from one in *pepQ* (bedaquiline) and *panD* to 1737 for *gid*. The genes with the highest number of frameshifts or premature stop codons included *gid* (1737), *ethA* (1259), *ald* (955, cycloserine), *pncA* (723), *eis* (275, kanamycin), *katg* (140), *mmpR5* (90, bedaquiline/clofazimine), *embR* (87, ethambutol), *thyA* (75), *mshA* (48, ethionamide) and *ddn* (40, delamanid) (Table S3, Fig. 1). An association analysis of phenotypic drug resistance and LOF variant counts aggregated to a gene level revealed 10 loci with significant associations (*P*<0.02; odds ratios>2; Table 1), including *katG*, *ethA*, *thyA*, *pncA*, *gid*, *tlyA* (capreomycin), *mmpR5*, *ald* and *mshA*. For each locus, a similar analysis by LOF variant type is presented (Table 2). Five established drug resistance loci (*ethR, eis, embR, rpsA* and *ahpC*) did not show any significant associations (*P*>0.05).

**Table 1.** Significantly associated loss of function (LOF) mutations* in drug resistance genes

Drug	Gene	Sensitive	Resistant	Odds ratio	95% CI	*P*-value	Other DR variant (%)†
PZA	*pncA*	31	334	57.8	39.87–83.77	<10^−185^	0
INH	*katG*	10	126	23.11	12.13–44.03	<10^−41^	38.2
STR	*gid*	179	189	2.00	1.62–2.47	<10^−27^	9.2
ETH	*ethA*	170	182	2.83	2.25–3.57	<10^−19^	23.3
CAP	*tlyA*	6	30	25.36	10.50–61.26	<10^−16^	2.8
PAS	*thyA*	2	9	53.66	11.43–251.97	<10^−7^	54.5
CFZ	*mmpR5*	1	4	86.37‡	9.33–799.24	0.0002	0
ETH	*mshA*	0	8	35.72‡	2.05–622.80	0.0004	62.5
BDQ	*mmpR5*	1	2	786.00‡	35.39–17 457.22	0.0014	0
CYS	*ald*	3	5	10.75‡	2.54–45.45	0.0174	0

*Frameshift, large deletion and stop codons.

†Percentage of samples with a drug susceptibility test (DST) result and an LOF mutation, which also have another known resistance variant.

‡Odds ratios may not be reliable due to small numbers of isolates with mutations.

BDQ, bedaquiline; CAP, capreomycin; CFZ, clofazimine; CYS, cycloserine; DLM, delamanid; DR, drug resistant; ETH, ethionamide; INH, isoniazid; PAS, *para*-aminosalicylicacid; PZA, pyrazinamide; STR, streptomycin.

**Table 2.** Contribution of known loss of function variant types

Drug	Gene	Types	Sensitive	Resistant	Odds ratio	Odds ratio 95% CI	*P*-value	%
PZA	*pncA*	Frameshift	22	218	50.20	32.30–78.02	<10^−18^	13
PZA	*pncA*	Large deletion	7	63	42.45	19.42–92.82	<10^−18^	3
PZA	*pncA*	Stop gained	1	38	177.34	24.34–1292.34	<10^−18^	2
CAP	*tlyA*	Frameshift	6	30	25.36	10.50–61.26	<10^−18^	5
INH	*katG*	Frameshift	6	63	19.07	8.25–44.08	<10^−18^	1
INH	*katG*	Large deletion	0	34	122.99	7.54–2006.94	<10^−15^	0
ETH	*ethA*	Frameshift	143	146	2.58	2.01–3.31	<10^−15^	22
STR	*gid*	Frameshift	19	47	4.58	2.68–7.82	<10^−11^	6
PAS	*thyA*	Large deletion	1	4	45.33	5.02–409.51	<10^−10^	7
STR	*gid*	Large deletion	35	51	2.69	1.75–4.15	<10^−7^	3
INH	*katG*	Stop gained	0	10	36.03	2.10–617.01	<10^−6^	0
CYS	*ald*	Frameshift	3	4	8.54	1.89–38.54	0.0008	37
ETH	*ethA*	Large deletion	3	5	3.70	0.88–15.52	0.06	2
ETH	*ethA*	Stop gained	6	2	0.74*	0.15–3.65	0.71	0
CAP	*tlyA*	Stop gained†	0	0	–	–	–	0
PAS	*thyA*	Stop gained†	0	0	–	–	–	1
BDQ	*mmpR5*	Frameshift†	0	0	–	–	–	100
CFZ	*mmpR5*	Frameshift†	0	0	–	–	–	100
DLM	*ddn*	Stop gained†	0	0	–	–	–	67
DLM	*fbiA*	Stop gained†	0	0	–	–	–	33

*Low odds ratio could be due to measurement error of the drug susceptibility test (DST) as *ethA* is a known activator of ethionamide and stop gained mutations will probably result in resistance.

†Due to low numbers of isolates with DSTs for BDQ, DLM and CFZ, a statistical assessment of association could not be performed.

BDQ, bedaquiline; CAP, capreomycin; CFZ, clofazimine; CYS, cycloserine; DLM, delamanid; ETH, ethionamide; INH, isoniazid; PAS, *para*-aminosalicylic acid; PZA, pyrazinamide; STR, streptomycin.

To characterize the contribution of LOF variants to resistance and their potential to improve current genotypic predictions, their prevalence (%) relative to all known drug resistance-conferring mutations was calculated. Frameshifts were found to contribute significantly, with the highest percentages reported for bedaquiline (100%), clofazimine (100%), cycloserine (36.7%), ethionamide (22.2%) and pyrazinamide (12.8%) resistance. Premature stop codons contributed significantly to delamanid (66.7%) resistance. Large deletions were rarer, although still significant with the highest percentage observed for PAS resistance (7.1%, Table 3).

**Table 3.** Contribution of different mutation types to drug resistance

Drug	Total variants*	No. of unique variants	SNP (%)	Frameshift (%)	In-frame indel (%)	Large deletion (%)	Stop gained (%)
Rifampicin	8827	94	99.5	0.0	0.5	0.0	0.0
Isoniazid	11288	231	98.5	0.9	0.0	0.4	0.1
Ethambutol	6787	58	100	0.0	0.0	0.0	0.0
Pyrazinamide	4589	512	80.7	13.3	0.5	3.5	2.1
Streptomycin	6349	60	91.5	5.9	0.0	2.6	0.0
Fluoroquinolones	2075	34	100	0.0	0.0	0.0	0.0
Amikacin	1273	5	100	0.0	0.0	0.0	0.0
Capreomycin	1382	32	95.1	4.8	0.0	0.0	0.1
Kanamycin	2116	12	100	0.0	0.0	0.0	0.0
Ethionamide	4594	292	74.8	23.2	0.0	1.6	0.4
PAS	581	48	91.7	0.0	0.0	7.1	1.2
Cycloserine	300	14	63.3	36.7	0.0	0.0	0.0

*Across 32 689 samples.

indel, insertion or deletion; PAS, *para*-aminosalicylic acid.

## Discussion

Whilst the role of SNPs in *M. tuberculosis* drug resistance has received much attention [4, 14], the prevalence and contribution of probable functional LOF mutations (deletions, frameshifts or premature stop codons) is less well understood. Many of these LOF variants are found in drug activators such as *pncA* and *ethA* and the methyltransferase *gid*, which had the highest number of all three variant types. Loss of function of *gid* has been associated with low-level resistance to streptomycin, and we estimated they represent ~9% of all streptomycin resistance variants found. The *pncA* gene codes for the pro-drug activator for pyrazinamide, with established strong evidence of resistance links [31], and we estimated that ~18% of all pyrazinamide-resistance variants found in the current study induced a loss of function. Ethionamide resistance can be conferred by disruptions in the pro-drug activator coded by *ethA,* and this locus had the highest proportion of LOF resistance variants (24%). Our analysis suggests a highly significant association between LOF mutations in *mshA* and resistance. A bio-activation pathway for ethionamide involving the MshA protein [32] has been proposed, supported by *in vitro* work revealing resistance and an enrichment of non-synonymous mutations in the *mshA* gene. Although only 46 *mshA* variants were observed, our findings suggest that the locus should be considered when predicting ethionamide resistance. While significant associations were found between LOF mutations and several drugs, penetrance is not always complete. This observed incomplete penetrance could potentially be due to the unreliable nature of phenotypic susceptibility tests for some drugs (e.g. pyrazinamide [33]) or low-level resistance being conferred (e.g. *gid* mutations for streptomycin resistance [34]).

While pipelines to call SNPs and small indels are well established, large deletions are more difficult to detect and require analyses that are often not standard in resistance prediction algorithms. Although relatively rare (2% of total variants), these variants represent a non-trivial contribution to resistance with significant proportions of PAS (7.1%), pyrazinamide (3.5%), streptomycin (2.6%) and ethionamide (1.6%) resistance variants detected. As expected, LOF mutations were only found in non-essential genes where the loss of function can still be tolerated, albeit at a probable fitness cost. Based on this observation, it is likely that detecting deletions and other LOF mutations will be important to resistance prediction to any current and future drug which is activated or modulated by a non-essential protein.

Interestingly, relatively high numbers of deletions and other LOF mutations were found in candidate resistance loci for the newer drugs bedaquiline and delamanid. While the number of isolates with susceptibility test data for these drugs was low, a highly significant association was found between the *mmpR5* gene and resistance to bedaquiline, which is consistent with experimental evidence [35]. An association was also found between *ddn* and delamanid, but only a low number of samples have the resistance phenotype, and functional validation is required. The exact contributions of deletions towards resistance for delamanid and bedaquiline are difficult to estimate, due to the recent timescales at which the drugs were introduced, the probable low levels of resistance in our dataset, and the lack of understanding of resistance mechanisms. However, the presence of these variants does sound alarm bells and the high number of large deletions in these genes relative to the low level of resistance highlights the need to detect these variants. Such insights will enhance genotypic resistance prediction, which is increasingly being used to inform clinical and infection control decision-making in high-TB burden countries.

## Supplementary Data

Supplementary material 1

Supplementary material 2
