# Supplementary material for: Whole genome sequencing reveals large deletions and other loss of function mutations in Mycobacterium tuberculosis drug resistance genes
Source: Microb Genom. 2021 Dec 10;7(12):000724. doi: 10.1099/mgen.0.000724 (PMC8767347; doi:10.1099/mgen.0.000724)

S1 Table
Summary of *Mycobacterium tuberculosis* isolates with sequencing data

Lineage	Sensitive	Pre-MDR	MDR	Pre-XDR	XDR	Other DR	Total
1	2337	390	232	34	6	156	3155
2	3638	928	1417	1160	585	532	8260
3	2610	382	350	160	49	194	3745
4	11548	1281	2127	820	364	560	16700
5	170	21	41	3	-	18	253
6	129	9	5	-	-	5	148
7	49	-	-	-	-	3	52
9	2	-	1	-	-	0	3
M. bovis	45	-	-	1	2	325	373
Total	20528	3011	4173	2178	1006	1793	32689

MDR = Multi-drug resistant, XDR = Extensively drug resistant, Other DR = drug resistant isolates which do not fall into other resistance categories

S2 Table
Resistance profiles of individual drugs

Drug	Sensitive	Resistant	Total
Rifampicin	12248	5436	17684
Isoniazid	11266	6263	17529
Ethambutol	12934	3163	16097
Pyrazinamide	10427	2272	12699
Streptomycin	4819	2640	7459
Ofloxacin	2641	845	3486
Kanamycin	2353	715	3068
Capreomycin	2405	503	2908
Amikacin	2187	473	2660
Ethionamide	1565	709	2274
Moxifloxacin	1413	300	1713
PAS	1123	103	1226
Cycloserine	964	154	1118
Clofazimine	584	31	615
Ciprofloxacin	337	65	402
Bedaquiline	394	3	397
Delamanid	393	4	397

PAS = para-aminosalicylic acid

S3 Table
Loss of function variants in the 23 candidate resistance genes*

Gene	Drug	Large deletions	Median deletion size (bp)	Frameshifts	Stop codons	Total
gid	streptomycin	165	131	1483	254	1902
ethA	ethionamide	72	1142	1021	238	1331
ald	cycloserine	5	2508	948	7	960
pncA	pyrazinamide	158	2201	585	138	881
eis	kanamycin	24	3597	235	40	299
katG	isoniazid	50	8748	103	37	190
thyA	PAS	41	5614	22	53	116
embR	ethambutol	27	846	79	8	114
mmpR5	CFZ/BDQ	3	564	87	3	93
tlyA	capreomycin	-	-	66	3	69
mshA	ethionamide	2	3074	44	4	50
ddn	delamanid	5	498	15	25	45
rpsA	pyrazinamide	-	-	40	-	40
ethR	ethionamide	29	2081	2	2	33
fbiC	delamanid	-	-	13	3	16
ahpC	isoniazid	4	2947	8	-	12
ribD	PAS	-	-	4	6	10
fgd1	delamanid	-	-	4	-	4
alr	cycloserine	-	-	-	4	4
rpsL	streptomycin	-	-	2	-	2
fbiA	delamanid	-	-	1	1	2
embB	ethambutol	1	55	-	-	1
pepQ	BDQ/CFZ	-	-	1	-	1
panD	pyrazinamide	-	-	1	-	1

* github.com/jodyphelan/tbdb; PAS = para-aminosalicylic acid; BDQ bedaquiline; CFZ clofazimine

Supplementary Figure 1
A map showing the locations where the isolates were sampled

Coloured countries indicate that samples were collected in the respective country. Darker shades indicate more samples originating from the country. Good geographical coverage is seen in the dataset with samples originating from all continents.

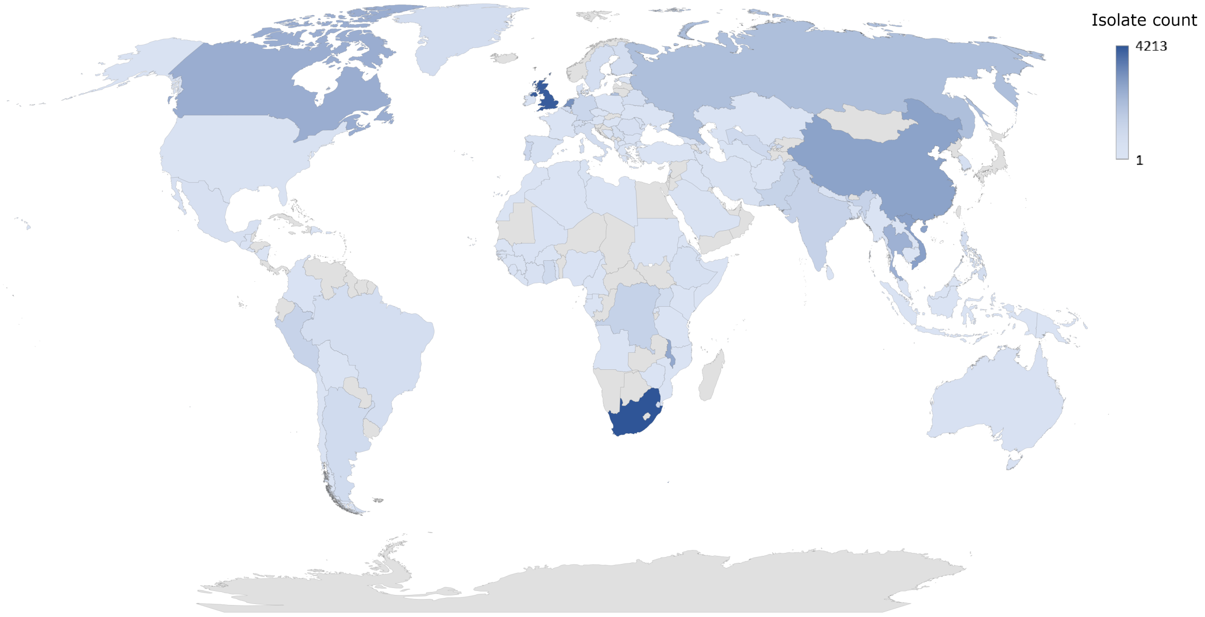

Supplement: Supplementary material 1 [file mgen-7-0724-s001.pdf]
